# The man behind the DNA fingerprints: an interview with Professor Sir Alec Jeffreys

**DOI:** 10.1186/2041-2223-4-21

**Published:** 2013-11-18

**Authors:** Alec J Jeffreys

**Affiliations:** 1 Department of Genetics, University of Leicester, Leicester LE1 7RH, UK

## Abstract

In this interview we talk with Professor Sir Alec Jeffreys about DNA fingerprinting, his wider scientific career, and the past, present and future of forensic DNA applications.

The podcast with excerpts from this interview is available at: http://www.biomedcentral.com/biome/alec-jeffreys.

## Introduction

Professor Sir Alec Jeffreys studied biochemistry and genetics at The University of Oxford, UK. Following an EMBO Postdoctoral Fellowship at the University of Amsterdam, The Netherlands, where he was one of the first to discover split genes, he moved in 1977 to the Department of Genetics at the University of Leicester where he held the positions of Professor of Genetics and Royal Society Wolfson Research Professor until September 2012 when he retired from research.

**Figure 1:**
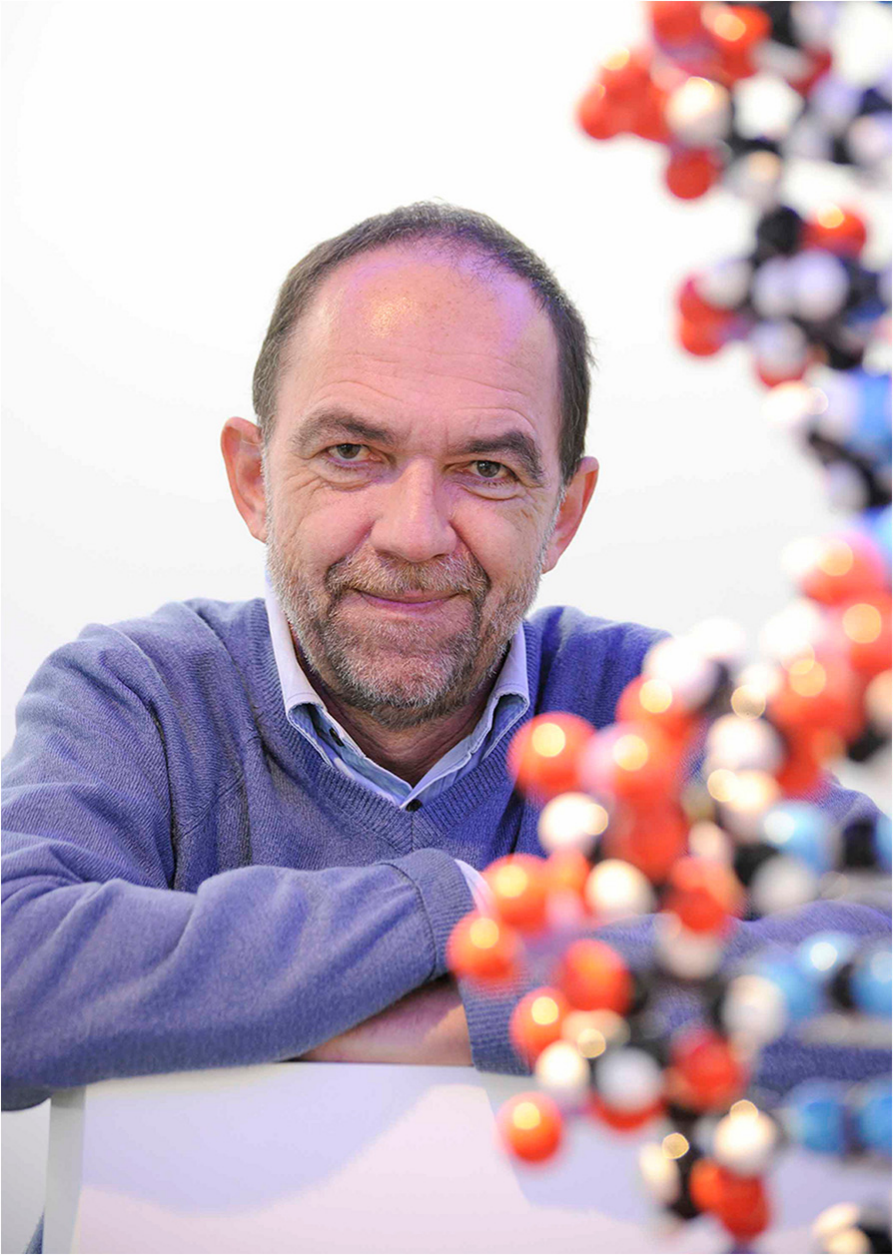
Professor Sir Alec Jeffreys (picture taken by Colin Brooks, courtesy University of Leicester).

Professor Jeffreys’ research at Leicester focussed on exploring human DNA diversity and the mutation processes that create this diversity. He was one of the first to discover inherited variation in human DNA, then went on to invent DNA fingerprinting, showing how it can be used to resolve issues of identity and kinship and beginning the field of forensic DNA.

## Transcript

### You are widely considered the founding father of DNA fingerprinting in general and forensic DNA identification in particular. Could you briefly describe what led you to the discovery of DNA fingerprinting?

Pure accident and academic curiosity, simple as that. If you wind the clock back to 1977–1978, the very first tools were emerging that enabled us to start exploring genetic variation in DNA sequences between people. That was restriction fragment length polymorphism (RFLP) technology, which was incredibly archaic and extremely tedious. I think we had our first RFLP in 1978. The issue then was that the markers that we were picking up were single nucleotide polymorphisms (SNPs) with very limited variability. So we felt that there was going to be considerable mileage for basic human genetics in developing markers that were much more variable between people. Intuitively, I felt that the sort of sequences you should be looking for would be tandem repeat DNA–what we now call minisatellites.

We started a research programme, I guess in about 1980, to try and isolate human minisatellites from the genome, and after all sorts of false starts we accidentally stumbled upon the fact that there is a short sequence shared amongst a considerable number of human minisatellites. That sequence then opened up the possibility of mass isolation of highly variable tandem repeat markers.

The driving force behind all this work was first of all academic curiosity, more than anything else. But secondly, a desire to be able to develop genetics markers far more informative than SNPs, for general use in the human medical genetics community. The last thing on our mind was forensic identification. That never occurred to us, and I think not to anybody else in the field at that time. Forensic DNA simply wasn’t on anybody’s radar.

In September 1984, we did the key experiment, which was to test whether a hybridisation probe, consisting of the little shared sequence tandem-repeated, could effectively detect multiple minisatellites in human DNA. That, completely by accident, led to what proved to be the world’s first DNA fingerprint–essentially a completely individual specific pattern. It was only after we got that first DNA fingerprint, or set of fingerprints, that the penny dropped that we’d accidentally stumbled upon a method for individual identification, and also for looking at family relationships. I think the penny dropped within about a minute of developing that first x-ray film. So this was a very exciting moment where, literally, my entire life changed in the space of about 60 seconds.

### At what point, and what were the indicators, that led you to realise the full potential of your new discovery?

It was pretty well instant. It was around about nine o’clock on a Monday morning that we got this result in. Within the hour we could see things like paternity testing, determining whether twins are identical or not and forensic investigations. We could even see the potential for non-human applications because on that first DNA fingerprint experiment we had included several non-human species. Most worked very nicely as well, so the potential was there for investigating wildlife crime and the genetic structure and biodiversity of wild populations. The one application I didn’t spot, and that was spotted by my wife that evening, was the potential use in immigration disputes where there are doubts about claimed family relationships. I have to say well done to my wife Sue because the very first casework ever taken on using DNA fingerprinting was an immigration dispute.

On that Monday, in the afternoon, we’d got our ideas together to the point where I was actually running round the lab pricking my finger and leaving blood spots all over the place–a sort of mock “scene of crime”, shall we say–because at that point we had no idea whether DNA even survived in forensic samples, never mind if it was amenable to DNA fingerprint analysis. So that Monday was pretty exciting.

### In addition to forensics, which other fields were immediately impacted by the discovery of DNA fingerprinting?

I think the big one was the entire field of molecular ecology: going out into the wild, using DNA fingerprinting to examine the genetic structure of wild populations. If you stop and think about it, that lies right at the heart of Darwinian evolution. Darwinian evolution is essentially survival of the fittest offspring. To understand that properly you have to know relationships: offspring of whom? Who are the parents? Very quickly there were colleagues of mine, particularly in the bird field, that took on DNA fingerprinting very actively indeed. They came up with fascinating observations, such as how the house sparrow indulges in spouse swapping, as revealed simply by looking at parentage of fledglings in a nest.

### In addition to forensics, which fields do you think have been impacted the most by the discovery of DNA fingerprinting?

Paternity testing, there’s absolutely no question about it. If you look at the status of paternity testing before DNA arrived on the scene, it was all determined by a battery of blood group and enzyme markers. They were quite effective and you could get indications of certainty up to roughly 99–99.9%. But of course, that means that there would be a significant error rate and if you happen to be the non-father who fails to be excluded as father of the child, that man unjustifiably would be required to give maintenance support for an individual who is not his child. That would have happened in a significant number of cases.

### What was the reason to eventually move away from using minisatellites in DNA identification?

The whole method was based upon taking microgram quantities of human DNA, chopping it up with a restriction enzyme, running it through a gel, doing a Southern blot, and then hybridising with a radioactive or chemiluminescent probe. That really became the gold standard in forensic labs worldwide right up until the 1990s (and was also the technology employed by commercial labs round the world), but it required significant amounts of DNA and it was very slow and tedious, with limited sensitivity. So quite often you would start with a sample and it might be a week or two before you got a result back. There was an urgent need to come up with some sort of technology that would speed the whole process up and also enable automation much more effectively.

Of course, the solution came with the development of user-friendly PCR in 1988. It was immediately apparent that that was going to be the way forward. The real question was: what marker could you test, or what set of markers would you choose in forensic identifications? There were a number of options. Short minisatellites could be quite effectively amplified in their entirety and typed, and there were a number of groups around the world that pursued that line of enquiry. We developed a system that combined SNPs with minisatellites. Minisatellites contain repeat units that are, not identical; there are single nucleotide variations within the repeat array. That enables you to encode a minisatellite allele, not in terms of length, but in terms of the arrangement of repeats along the array. I have to say I’m very proud we developed quite a neat method for reading out these digital signatures of a minisatellite allele from DNA, using PCR. Mitochondrial DNA of course was very much in the focus, but being a maternally inherited marker, it failed to discriminate between close maternal relatives.

Another possibility was SNPs. Those are still very much in the frame, particularly with the advent of the latest sequencing platforms and SNP-typing platforms that can type huge numbers of SNPs at a time. But going back to the SNP-based PCR systems that were developed in the early 1990s, they were effective but based on exclusion rather than inclusion, having little power of discrimination.

The final system was a new type of marker first discovered almost immediately following the availability of PCR, namely microsatellites or short tandem repeats (STRs), where you absolutely require PCR to be able to type variation. Those were the way forward and, in fact, we got involved almost immediately in a programme first of all to isolate more of these markers, but secondly to actually deploy these in real forensic casework. One case we took on was the so-called Cardiff “body in a carpet” case, where someone had bought a house in Cardiff, dug up the patio, and found a carpet bound up with wire with a skeleton inside. That’s a clear sign of foul play, there’s no question about that. The $64,000 question was: who was the victim?

The skull was used as a basis for a facial reconstruction that was put out on UK TV, and someone phoned in and said “It looks rather like Karen Price,” who was a girl who had gone missing about ten years previously. Armed with that very provisional identification, a colleague and I were asked to attempt a bone DNA analysis to compare DNA from the skeletal remains with those of Karen Price’s mother and father. That’s what we did, and got a positive result with a level of certainty better than 99.9% that these really were the remains of Karen Price. That proved to be important evidence in the trial, which led to a double conviction.

The other case, which we initiated about the same time, was the identification of skeletal remains of Josef Mengele, the Auschwitz concentration camp doctor, that had been exhumed in 1985 from a gravesite in Brazil. Once again, the key question for this major war criminal was: was the identification correct or not? We were asked by a German prosecutor to carry out a full DNA analysis, comparing the remains with DNA from Mengele’s wife and son. And again using PCR and a battery of microsatellite markers, we got a positive identification with a level of certainty better than 99.9%.

That information, together with a lot of other information involving very careful investigation of facial features from the skull, and matching those with photographs of Mengele, allowed the prosecutors to come to the conclusion that it was a positive identification and the case against Mengele was closed. That was an important contribution in that the war crimes investigation against Mengele had been dragging on for about 40 years and was probably the biggest investigation ever mounted against an individual.

### Most of your scientific career was outside of DNA fingerprinting; can you summarise the most important outcomes of your research on the molecular basis of human genetic recombination?

Well, I always describe my career as a fairly logical progression from developing tools for detecting single-copy DNA sequences back in the mid-1970s right the way through to the latest recombination work, and then along that linear track there’s a huge great loop coming out the side and that’s forensic DNA, which started in 1984 and, for me personally, finished by the early-to-mid-1990s. My real interest was always in the fundamental biology of DNA.

Probably the most important thing I ever did was, following my DPhil in Oxford, to spend two years in Amsterdam in the mid-1970s with Richard Flavell. We attempted to isolate a mammalian gene, which had never been done before; we had no idea what genes looked like. It was during that work that we developed Southern blot methods sensitive enough to detect single-copy genes. So we could actually see a mammalian gene for the first time, by direct experimental analysis. That led, in 1977, to one of the first descriptions of introns in genes. Then in 1977 I moved to Leicester and was faced with a very interesting career choice: do I carry on with the intron work? I knew that was not going to be sustainable since there was going to be an army of very well-resourced labs moving into that field. So I decided to combine the ability to detect single-copy gene sequences with my exposure to basic human genetics as a doctoral student. Put the two together and see if you can find RFLPs. Within less than a year of arriving in Leicester, I’d set up a lab and was getting our first RFLPs coming off the line. That I think was a very important contribution.

Also, I was very proud of the fact that I used what proved to be an incredibly small survey to come up with the first estimate of the total number of SNPs in the human genome. The number we came up with was 30 million. A friend of mine, Peter Little, pointed out that I’d messed up the calculation (we’re all human), and the real number was 15 million. Of course, that was pretty much spot on the number of SNPs that have now been revealed through genome sequencing. That was the first estimate of how many SNPs you might find in human DNA.

Then we moved on to the minisatellite work and the applications. But behind that forensic work, we still kept a very active programme going of asking very basic questions. Minisatellites are fantastically variable; some of these have, if you use the right discrimination system, millions, if not billions, of different alleles worldwide. That variability means one thing only: that they must have a high rate of germline instability and are mutating very rapidly, adding and losing repeat units and creating new alleles.

The obvious question then was: what is the mechanism of tandem repeat instability? The two broad possibilities were, first, instability driven by aberrations in DNA replication or repair; and second, recombination at either mitosis or meiosis. So we set about first of all directly measuring mutation rates of human minisatellites, and we succeeded in that I think in the late 1980s. So for the first time, you could directly measure a germline mutation rate in humans.

Then the question was, what is the process? Using the detailed structural information we could get on minisatellite alleles before and after mutation, it became very clear that the dominant mutation process was meiotic recombination, leading to transfers of repeat units from one allele to another, in a sort of gene-conversion-like process.

The next question was, if the driver is meiotic recombination, is the instability an intrinsic feature of a minisatellite repeat array, or is it driven by some external element which is forcing the array to become unstable? We wanted to know if we could detect genuine, regular meiotic crossover events. So we developed a whole raft of single DNA molecule methods whereby we could pan huge numbers (millions) of sperm for what were likely to be very rare events going on near minisatellites. That went very well and led to the first full molecular description of a meiotic recombination hotspot. In fact, for the minisatellite we were investigating, there was a hotspot located right next to the minisatellite, about a kilobase and a half wide.

Now, hotspots had been hypothesised in human DNA for decades previously, with DNA diversity evidence suggesting that there might be a focussing of DNA recombination events preferentially in hotspot regions, but this was a first, clear demonstration of exactly what a hotspot looked like: a bell-shaped curve of recombination activity near this minisatellite. The intriguing thing was that the hotspot was not inside the minisatellite, but centred next door to the minisatellite. That, along with a number of other lines of evidence, suggested it was that hotspot that was driving instability in the minisatellite. In other words, the minisatellite was acting as if it was a parasite of meiotic recombination hotspot activity.

Now we knew that there was a connection between minisatellites and recombination, the next question was are the sort of hotspots that you see near minisatellites a more general feature of the human genome or something obscure only found in minisatellites? We then embarked in the year 2000 on a systematic analysis of this, using again some challenging single-sperm techniques to directly measure male recombination frequencies at intervals across selected areas of the human genome, the main focus being on a region within the major histocompatibility complex. And what that showed was that recombination hotspots 'rule the roost’; that the great majority of meiotic recombination events in human DNA focus into hotspots and that these hotspots have a huge impact on patterns of human DNA diversity. Between the hotspots, DNA rarely recombines. There are very strong associations between all markers within these regions and they behave as so-called haplotype blocks, blocks of markers that have historically not been reshuffled by meiotic recombination. Rather curiously, even though recombination is an astonishingly important process in terms of driving human DNA diversity, most of the human genome seems to not be aware it’s in a sexual organism and is not subjected to meiotic recombination reshuffling.

The next questions were based on the properties of recombination hotspots. We quickly discovered that there were some odd things going on in these hotspots. In particular, they had a propensity to destroy themselves. If a mutation arises in a hotspot that downregulates the initiation of recombination in the hotspot, then the mutation will tend to be over transmitted to recombinant progeny and will sweep through a population. This was meiotic drive, with the meiotic drive element being a single-base variant, which had this ability to sweep through a population and shut down a recombination hotspot. Other people had noticed similar things in other organisms, including Rosie Redfield who first articulated what was going to be a big problem: how do hotspots exist if they commit suicide? This was the so-called hotspot conversion paradox, which remained unresolved for a good number of years.

The next and final phase of my research life was facing the big question of what on Earth is specifying hotspots and how come they can survive in the face of this suicidal tendency. The answer to that came in 2010, from three labs who independently identified a gene called PRDM9 as the likely specifier of hotspot locations in the human and the mouse genomes. Having seen this, we immediately thought: “we can take people with different PRDM9 genotypes and find out if people with variant versions of PRDM9 differ in their hotspot recombination activity from people carrying the standard European allele”. It very quickly became apparent that yes they did and PRDM9 really was *the* major specifier of recombination hotspot activity and location, and did so by virtue of a tandem repeat zinc finger array within the protein recognising a short DNA sequence motif associated with hotspots that had been noted by bioinformaticians.

Of course, this was very exciting for me because the PRDM9 gene contained within it a classic minisatellite coding for this tandem-repeat zinc finger array. So I’d come suddenly full circle, right the way back into the world of minisatellites. The hotspot conversion paradox is then in principle neatly solved. Hotspots don’t hang around, they don’t persist; they do commit suicide, but are being continuously topped up by mutations within this minisatellite creating new versions of PRDM9 that can recognise new sequence motifs and specify new sets of hotspots.

The final act of my career was to directly analyse levels of instability at the PRDM9 minisatellite to see whether the theory would work, and the answer is that the model most certainly does work; PRDM9 alleles cannot survive more than maybe a million years or so before vanishing. In other words, the recombination landscape itself, right across the genome, is highly turbulent.

The final little twist to the story was that PRDM9 influences not only recombination hotspot activity, but also unequal crossing-over events, which was another major research programme in my laboratory; we showed for one particular class of unequal crossover event that generates pathological rearrangements in the human genome that again these events only occur in people with the appropriate PRDM9 allele, in other words, a different type of PRDM9 variant can act as protective against these *de novo* rearrangements. So we have PRDM9 controlling hotspot locations and influencing at least some classes of unequal crossover in the human genome, but it also turned out that the PRDM9 protein influences the instability of its own coding sequence, which was really bizarre. You then have this scenario where you can in theory have PRDM9 variants appearing in a population that heavily destabilise their coding sequence, leading to an explosion of new mutations and sudden burst of new PRDM9 variants out there in the population. But actually–as it turns out from simulation work–this is a highly self-limiting process, so you wind up with a totally bizarre situation whereby the level of diversity of PRDM9 in the population tends to be determined mainly by the instability of the least unstable variants in the population, not the most mutable variants. At that point, I felt it was time to retire, so I retired.

### You’re extremely passionate about genetics and its impact on society, as well as conveying science to the general public including today’s youth. Can you describe your motivations in this regard, and any particularly memorable experiences?

The motivation is just one of excitement; essentially, if you cannot sell science on the back of rape, murder and mayhem, which is basically the story of forensic DNA, you might as well give up. I give talks to schoolchildren, lay audiences and so on, and it really is an easy sell. The important thing is that you can get a lot of the basic science across on the back of casework. My feeling is that if I can enthuse one school child to get excited about science and maybe follow a career in science in the future, that makes it all worthwhile. I find it enormously satisfying just to see an audience get switched on by this sort of forensic DNA work.

The downside is that if you ask kids what they want to do then they’ll say they want to be a forensic scientist. Sadly, there are rather limited opportunities in the forensic science field for new employment. For example, we’ve seen the present government wind up the entire Forensic Science Service. There is virtually no public sector presence in forensic science now; virtually all the testing is done in the commercial sector. Every kid wants to be a forensic scientist out there doing forensic DNA, but if that enthusiasm can be diverted to enthusiasm more generally for chemistry or biology that’s absolutely terrific.

In terms of notable public engagements, I’ve done a lot of TV and radio appearances. For example, I think one of my favourite occasions was the Daily Mirror Pride of Britain ceremony, where I received a lifetime achievement award. Most awardees would have a guest appearance, and for mine they found Kirk Bloodsworth, who was the first person to be exonerated by DNA in the United States. He was convicted of the rape and murder of a 9-year-old girl around the same time that we came up with our first DNA fingerprint. He was convicted and wound up on Death Row facing execution in the gas chamber. He happened to read about DNA and thought “This is a possible way forward”, and after a great battle he eventually got the DNA testing results in and was exonerated. They later identified the true perpetrator. Meeting him was a really emotional moment.

### Forensic DNA databases are an instrumental tool for developing investigative leads. Do you see any downside to their use?

In short, yes. DNA databases are spreading very actively. Most major countries have implemented a national criminal intelligence DNA database, and the first of course was the UK with a database, which, per capita, still remains the largest in the world. It has been very actively populated over the years, starting in 1995. It has had a very significant impact in the fight against crime; for example, if you can find a crime scene DNA sample, in better than 50 per cent of cases this will give a match on the database and a lead towards a prime suspect. That can short circuit a huge amount of police investigation.

On the downside, there are some specialist applications, for example familial searching where you may have a crime scene sample where you don’t get a full match in the database but instead a partial match, perhaps pointing to a relative of the true perpetrator. I think there are some ethical issues that still haven’t been fully resolved, bearing in mind that familial searching is not to my knowledge governed by any legislation. It’s pretty much a free-for-all as to whether you want to do it or not.

I think the bigger issue was a change in the UK law back in 2003 that allowed police to obtain and retain DNA from anybody they arrested, irrespective of whether there was subsequent charging or conviction. Now that led to roughly one and a half innocent people being added to the database. That became blatantly discriminatory. I’ve seen claims of in excess of 80 per cent of juvenile male blacks in the UK now residing on the database. This is basically branding an entire section of society as 'if they’re not a criminal now, they’re going to be criminal in the future’. Anyway, that retention was declared illegal a number of years ago by the European Court of Human Rights and also by the UK Supreme Court, and legislation in the UK has been implemented to remove these innocents from the database. The only exception is in the case of people arrested with respect to very serious crimes, such as rape and murder, where I think there is a reasonable argument for retaining, in a time-limited fashion, their DNA profiles.

### If you were to construct legislation on forensic DNA databases, what constraints would you put in place?

Well I would be pretty content with keeping things much as they are at the moment, so that the police can retain a database from convicted individuals. If you’re convicted of a crime, you owe it to society to have your DNA retained so that in the event of re-offending you’re picked up quickly. And it should be on a limited set of genetic markers that carry little information on you as an individual, in particular in terms of future disease liability.

But for the police to switch platform to routinely sequencing the entire human genome would raise massive issues in genetic privacy, basic questions about who owns your genome. I take the very simple view that my genome is my personal property and is not up for grabs nor is available for free and open viewing by any organisation. There are many tensions between genetic privacy and the state’s desire to minimise crime and to maximise health, and I think we’re still very much at the beginning of that debate.

### How do you see science and technology of forensic DNA applications moving forward in the next five to ten years?

I never engage in crystal ball gazing because you just don’t know what’s around the corner. If I’d answered this question on blood groups in 1980, my answer would’ve been 100% incorrect because we would never have seen DNA fingerprinting coming over the horizon.

First of all, will next-generation sequencing impact forensic investigation? Will there be a wholesale move away from the current STR-based typing systems to something far more sophisticated reflecting modern technology rather than technology that is now about 20 years old? My guess is probably no. It's fairly straightforward to take STR evidence to court. You can explain what the profiles look like and get the jury to interpret, but taking a few terabytes worth of DNA sequence information to court and throwing that at a jury will encounter problems. However, you can see future specialised applications of next-generation sequencing; for example, in the event of bioterrorism it would be possible to search the scene for evidence of a known biological weapon or biological agent that can be weaponised.

Another big area is the notion of predicting phenotype from DNA sequence variation. That’s particularly important for police when they have a crime scene DNA sample and no suspect. This is a vast area that ranges from trying to predict phenotypes such as hair and eye colour from DNA sequences all the way through to ancestry predictions and taking DNA variants and trying to determine the geographical origins of an individual. These applications are all looking pretty good at present.

There are some interesting new developments in the field that appear to be able to carry out really quite fine geographical discriminations. For example, it seems to be quite possible to distinguish people from the Orkney Islands from the rest of the UK. This is an area that I think is absolutely fascinating for genealogy, since everybody wants to know their origins and family history. But whether this is appropriate for police work, again there are ethical issues about genetic privacy that may come into play here. For example, if you’re looking at genes that control facial features, I think we can absolutely guarantee that some of the key genetic variants will carry congenital abnormality risk information with them. Should the police be accessing that type of information? If they do get that information, what should they do? If they’ve identified someone who's brought into a crime scene investigation, are the police required to say “OK, yes, we’ve brought you in to this investigation, we can actually acquit you of this crime, but by the way you carry 'x’ mutation and you should be aware that your family’s at risk of 'x’ disease”.

That really raises some pretty serious questions about who should have access to this sort of information. I see that sort of phenotypic prediction developing a lot, but I think probably largely driven by academic curiosity rather than by an overarching need to develop sophisticated phenotypic predictions for police investigation work. The alternative, of course, in police investigations is to just stick everybody on the DNA database. The United Arab Emirates enacted legislation for exactly that quite some time ago for the mandatory databasing of the entire 10 million population. This is a very interesting social experiment and I’ll be very interested to see how it plays out.

## Competing interest

AJ has no competing financial interests to declare.

